# Understanding Co‐Creation in a Research Partnership Programme Exploring Patient‐Driven Innovations: A Qualitative Longitudinal Study

**DOI:** 10.1111/hex.70003

**Published:** 2024-08-30

**Authors:** Hanna Jansson, Jamie L. Luckhaus, Henna Hasson, Pamela Mazzocato, Terese Stenfors, Carolina Wannheden

**Affiliations:** ^1^ Department of Learning, Informatics, Management and Ethics, Medical Management Centre Karolinska Institutet Stockholm Sweden; ^2^ Center for Epidemiology and Community Medicine (CES), Region Stockholm Stockholm Sweden; ^3^ Research, Development, Education, and Innovation Unit Södertälje Hospital Södertälje Sweden; ^4^ Department of Learning, Informatics, Management and Ethics, Division of Learning Karolinska Institutet Stockholm Sweden

**Keywords:** co‐creation, co‐production, partnership research, patient and public involvement, patient author, patient‐driven innovation

## Abstract

**Background:**

Research indicates that successful co‐creation depends on a shared understanding of co‐creation and its related concepts. However, it also shows that, in practice, views on co‐creation and how to do it differ. This study aims to explore how patient innovators and researchers in a partnership research programme understand co‐creation and how this understanding changes over time.

**Methods:**

An explorative longitudinal qualitative study was conducted with the ‘Patients in the Driver's Seat’ partnership research programme. Fifty‐eight interviews were performed and analysed using a reflexive thematic approach.

**Findings:**

Four different ways of understanding co‐creation were identified. These can be instrumentally conceptualized as themes using the *inputs‐process‐outputs* model: (1) combining different perspectives, experiences and backgrounds (*inputs*); (2) deliberately dynamic and exploratory (*process*); (3) striving for equity, not equality (*process*); and (4) diverse value creation, tangible and intangible (*outputs*). Together, these themes represent the varied understandings of co‐creation among partnership programme members.

**Conclusions:**

Our study of patient innovators and researchers identified four distinct yet complementary understandings of co‐creation. The study suggests that co‐creation is the sum of its essential components, which can be divided into *inputs*, *process*, and *outputs*.

**Patient or Public Contribution:**

This study, and the partnership programme it explored, aims to improve the relevance of research for patients and informal caregivers through an improved understanding of the concept of co‐creation within research on patient innovation. All patient innovators involved in the programme were invited, as interviewees and researchers, to contribute to the study design and data analysis.

## 1 Introduction

With advancements in research and innovation, improved diagnostic methods and treatments, people are living longer, despite more chronic illness. While patients are able to live longer with their conditions, many still struggle and want to improve their care and healthcare systems. They identify needs and generate new or improved approaches that are of practical use, initially for themselves. Although challenging, in the right context, their struggles and individual solutions can lead to innovations and benefit others as well. In previous studies, patients (or their non‐professional caregivers) who ‘modify or develop a treatment, a technical aid product or a medical device to cope with a health condition’ are called *patient innovators* [1]. On the website Patient Innovation (https://www.patient-innovation.com), a global community where patients and caregivers share their solutions to health‐related problems, over 1000 innovations are listed. Although growing in number, from a research perspective, the number of patient‐driven innovations published in peer‐reviewed journals is still quite small [2]. Studies of patient‐driven innovations that have been published indicate that innovations aim to improve access to self‐care support tools, open sharing of information and knowledge, as well as patient agency in self‐care and healthcare [3]. The research community, from researchers and journal editors to funders, needs to do more to involve patients in their research and publishing. Traditionally, this has taken the form of one‐directional patient involvement in research projects [4]. However, more recent literature suggests that research should be carried out in partnership with patients, their family members and academic researchers [5]. Patient innovators have reported positive experiences (e.g., mutual respect, meaningful participation, and learning and competence development) but also negative experiences (e.g., cultural and structural barriers, and power imbalances) in research collaborations [6]. Equal partnerships and co‐creation of research among all stakeholders (rather than one group simply providing feedback to others) are now considered essential.

### 1.1 Co‐Creation Research

Research into co‐creation is rooted in various fields and disciplines [7, 8] and has adopted different perspectives. Depending on the context and research focus, different terminology and frameworks are used [9]. At the core of co‐creation research, however, lies the assumption that a partnership between researchers (who know how to conduct research) and a partner with practical or lived experience can contribute to actionable and useable research and thus reduce the know‐do gap [10]. Co‐creation research typically implies involvement in all phases of the research process, from design and data collection to analysis and to publication [8].

The research suggests that co‐creation can be achieved through a variety of approaches. Many scholars describe co‐creation as co‐production between individuals from different organizations [11]. Others describe it as action research, where the researcher can participate in practical development work with practitioners [12]. From a participatory research perspective, the researcher invites practitioners to participate in a research project [13]. The level of engagement varies from sharing experiences to full participation on an equal basis [14].

The literature points towards several key factors that contribute to successful co‐creation. Among these factors are quality relations and trust [15]; the ability to expose and work through tensions; and the opportunity for co‐creation from the project planning stage onwards [16]. Co‐creation project teams often involve members with different experiences and knowledge. This heterogeneity can contribute to different understandings of core working concepts [17]. In studies on co‐creation in research, the concept as such as is often neither formally defined nor critically discussed [17]. Agreeing the meaning of concepts early on in a co‐creation process and developing a shared language is essential for group success [18], since members, even with similar backgrounds, can express themselves differently. Researchers may speak the same ‘language’, for example, but conflicts can still arise if they interpret a project's objectives and methods in different ways.

Despite the existing literature on co‐creation, there is a gap in understanding how this concept is perceived and enacted within specific contexts, such as research projects in partnership with patient innovators. The existing applications often provide broad, theoretical perspectives but may not capture the nuanced, context‐dependent understandings that emerge in practice. This underscores the need for a context‐specific exploration of co‐creation to understand how it is experienced by participants in real‐world settings.

## 2 Methods

### 2.1 Study Design and Setting

This is an explorative longitudinal qualitative interview study [19, 20] grounded in a social constructivist paradigm [21, 22] with the understanding that there is no universal definition of *co‐creation*. We rather accept that knowledge and meanings are co‐constructed through social interactions. Adopting a social constructivist viewpoint allows us to explore the concept of co‐creation as it is understood by patient innovators and researchers in a partnership research programme. This approach not only fills the research gap but also offers valuable insights into how co‐creation can be effectively fostered and supported in similar contexts.

The study followed the members of the ‘Patients in the Driver's Seat’ research programme based at the Medical Management Centre at the Karolinska Institutet in Sweden. This is a 6‐year partnership programme (2019–2024) funded by FORTE, one of the national research funders in Sweden. The programme builds on five medical innovations: Genia, Dream Catcher, CareMaps, Patient Recovery Education and Patient Lead Users. All were developed and driven by patients or informal caregivers to promote self‐ and co‐care. They are intended as a form of service aiming for social value, delivered using a variety of approaches, from low‐tech ‘pen and paper’ and digital applications to educational programmes and popular movements. The research programme studies their development and implementation into the health service and the daily lives of patients. Each innovation is represented in the programme by at least one patient or informal caregiver involved in its development, referred to as ‘patient innovators’.

The programme's ambition (as stated in the funding application) was to ensure that patient innovators are given the opportunity to be engaged as equitable partners throughout the research process alongside educated researchers. The term ‘partnership programme’ was used in the funding application with the intention of equal collaboration. However, there was no articulated definition of *co‐creation* or *partnership*. Principles for the programme were discussed and decided together within the first year of activity. These were agreed to be: (1) the programme is based on co‐creation; all members have the right to say when something does not feel co‐created; (2) the patient innovators are the ‘driving force’ within the group and have complete authority over their respective innovations; (3) researchers may only access the innovations and the healthcare settings that use them through the patient innovators; and (4) conflicting interests and opinions should be seen as learning opportunities. The programme was led by a principal investigator (PI) and a co‐PI. However, decisions were made by a ‘management group’ formed of an equal number of patient innovators and researchers.

### 2.2 Data Collection

Four rounds of interviews were performed with members of the partnership programme as study participants. The overarching aim of the interviews was to explore the members' experiences of the programme and its development over time. Therefore, the interview guide was broader than the specific scope of this study. The programme members jointly drafted and revised the interview guide between interview rounds (Appendix S1). The topic areas for this study were (1) understanding and (2) experiences of co‐creation within the programme. The focus shifted slightly between rounds, reflecting the progress in the partnership programme, starting with the background of participants, then the programme structure, examples of co‐creation and finally learnings.

Two external female research assistants trained in qualitative methods and interview techniques conducted the first three interview rounds. The fourth round was conducted by J.L.L., who had limited contact with the programme members and involvement in programme activities but had read all of the transcripts from the previous rounds. The first two rounds were conducted in person at the respondent's workplace (or by phone, as one respondent preferred) with only the interviewer and the interviewee present. Due to contact restrictions resulting from the COVID‐19 pandemic, the third and fourth rounds took place via a videoconferencing system. Programme members were allowed to have the interview conducted in English or Swedish according to their preference. This yielded four English interviews. The interviews lasted 15–90 (mean 45) min. The interviews were audio‐recorded and transcribed by an external transcription service (Rounds 1–3) and J.L.L. (Round 4).

The four interview rounds were conducted in February–March 2019, November–December 2019, October 2020 and November 2021–March 2022. Two exit interviews were conducted within a month of a member's last day in the programme and were added to the interviews of the nearest interview round. The interviewers invited, by email, all programme members (researchers, assistants and patient innovators) to participate in the study, regardless of their time contracted to the programme (full‐time, part‐time and temporary) or the nature of their position in the team (junior or senior). The membership of the programme changed across time, as individuals left and joined the group, which resulted in varying degrees of interview participation for programme members.

Fifty‐eight interviews were performed. Twenty‐four programme members (18 women and 6 men) participated in at least one of the interview rounds (Table 1). This included patient innovators (7), research assistants and doctoral students (3), and junior and senior researchers (14). Five members participated in all four interview rounds, seven participated in three rounds, eight participated in only one round, and the remaining 38 in two rounds. Study participants consisted of an interdisciplinary group with a variety of professional backgrounds, reflecting a heterogeneous sample.

**Table 1:** An overview of participants, that is, interviewed members of the partnership programme.

Interviews	Round 1	Round 2	Round 3	Round 4
Time conducted	Spring 2019	Fall 2019	Fall 2020	Fall 2021
Number of researchers	8	9	10	14
Number of patient innovators	4	5	3	5
Total participants	12	14	13	19
Average length	34 min	56 min	51 min	38 min

### 2.3 The Co‐Author Team

All authors except J.L.L. had dual roles as co‐authors and study participants as members of the partnership research programme, ‘Patients in the Driver's Seat’. J.L.L., who conducted the interviews for Round 4, was minimally engaged in other parts of the partnership programme to maintain an ‘outsider’ role and, as such, to handle the sensitive information forming the first step of the analysis.

### 2.4 Data Analysis

Interview data were analysed longitudinally using a reflexive thematic analysis approach, following Braun and Clarkes' six‐phase process [23, 24]. As this study was exploratory in nature, we chose an inductive approach so we could be open to any experiences and understandings expressed in the data and, rather than imposing the restraints of a deductive approach, so we could produce themes ‘grounded’ in the data [21, 23]. Furthermore, since the study was grounded in a social constructivist paradigm, data were analysed with attention to contextual factors influencing participants' understanding of co‐creation. Throughout the analysis, we remained attentive to the specific context of the partnership programme and the unique experiences of each participant.

Only one author (J.L.L.), who was not an interview respondent, analysed the raw data. Their role as an ‘outsider’ was deliberately created to handle potentially sensitive information. First, J.L.L. read through all the interview transcripts multiple times to become familiar with the data and selected all data extracts relevant to the research question (pertaining to co‐creation). Once these extracts were anonymized, H.J. and J.L.L. read through them together and further reduced or divided them into codes to maintain as much context as possible while having manageable and concise codes. The same two authors then developed themes by comparing and contrasting data extracts using the online visual collaboration platform Miro [25]. This analysis was done separately for each interview round to facilitate longitudinal comparison as a final step. Throughout the data analysis process, collaborative note‐taking was also captured in Miro to maintain reflexivity, acknowledge individual influence and ensure transparency between coders. Interview transcripts were studied both individually and alongside each other. English and Swedish interviews were analysed together in their original language.

Synthesized member checking [26] was conducted in a workshop (November 2022) to which all partnership programme members were invited, and multiple patient innovators participated. As part of the workshop, the initial clustering of data extracts was presented, where (in groups) all participants were asked to identify the ‘theme’ of each clustering and to move or remove extracts as they saw fit. They were also asked to discuss relationships between themes as a second step. Finally, the groups were asked to compare and contrast their rearrangements and overall reflections with the rest of the workshop group (an example is provided in Appendix S2).

After the workshop, H.J. and J.L.L. continued to refine and name the themes. All four rounds were compared (Appendix S3), with reference back to the data extracts to distinguish the difference between real change over time and natural differences that occur in phrasing across an iterative process. However, no significant differences in descriptions of co‐creation between rounds were identified in the longitudinal analysis. Lastly, when writing the manuscript, all authors contributed by relating the results to the study's aim and the existing research literature.

### 2.5 Ethical Considerations

Written and verbal informed consent were obtained before each interview. Participants were informed that the interview was optional and made aware of who would have access to their interview files. Ethical approval for the study was obtained from the Swedish Ethical Review Authority (Reg. No. 2019‐03849).

## 3 Findings

At its most basic level, participants described co‐creation as ‘doing together’. In the initial phase of the programme (Rounds 1 and 2), participants based their understandings of co‐creation on previous experiences and expectations, as well as on their aspirations for their work with the programme. In the later phases (Rounds 3 and 4), participants spoke about co‐creation from their experience within this particular partnership programme. The thematic analysis identified four ways of understanding co‐creation, or themes (Table 2). Informants could attribute multiple of the following themes to their individual understanding of co‐creation, and their understanding could also change over time. The illustrative quotations (I = patient innovator; R = researcher) were translated from Swedish to English with minor adjustments to enhance readability.

**Table 2:** A thematic overview with descriptions of understandings of co‐creation.

Theme	Co‐creation is understood as …
Combining different perspectives, experiences and backgrounds	… the combination of everything that is brought to the table by focusing on member differences
Deliberately dynamic and exploratory	… an open and explorative approach that allows all members to co‐create the entire process
Striving for equity, not equality	… co‐ownership which ensures equitable participation among all members
Diverse value creation, tangible and intangible	… the generation of value; tangible change, such as developments and scientific publications, and intangible, such as greater knowledge

### 3.1 Combining Different Perspectives, Experiences, and Backgrounds

This first theme describes members' understanding of co‐creation as the combination of everything that individual member differences (in background, perspective and experience) bring to the table. In line with the scope of the partnership programme, participants described co‐creation as collaborative work between patients, close relatives, researchers and innovators. The different perspectives were there to ‘complement each other’. As the programme progressed, participants began to describe co‐creation as collaborative work where different perspectives combined to create something bigger than the individual contributing parts.

It's great that so many of us come from different backgrounds. Hopefully we all want to hear these different perspectives as well. That's really co‐creation.

(R15, Round 4)

Members stated that involving people from different backgrounds makes co‐creation more ‘enriching’. It is important to ‘include the competencies needed to fulfil’ the aim of the partnership programme. This act of combining was described in the sense that co‐creation generates a broader perspective. One participant more specifically said that co‐creation is when you take in other perspectives but also ‘dare to question your own’. Combining was also described in terms of the intentionally interdisciplinary and diverse group of trained researchers involved. Co‐creation not only occurred between patient innovators and researchers but also among all programme members, including those from similar educational and professional backgrounds.

Everyone contributes with their competencies. We do it together … that everyone is included and contributes, that's where we get the different perspectives. Because, if someone becomes dominant, if it just becomes the researchers' perspectives, then it won't be co‐creation.

(R9, Round 4)

### 3.2 Deliberately Dynamic and Explorative

This theme refers to members' understanding that co‐creation involves an open and explorative approach, allowing all members to co‐create the entire process. The participants described a research process that was open to all members throughout the project's duration. They acknowledged that rules and routines were needed but said they should be co‐created (as part of the process) rather than set beforehand. If their programme had started with preset rules to follow, the participants felt it would not have been co‐created.

I don't think that you can just start to co‐create. Like, just ask how should we do it and then do it. Rather, it's something that we figure out as we go along, that now we need to do this. How will we go about it?

(R10, Round 4)

In the partnership programme, all the necessary conditions were in place: funding, cases to study and develop (innovations), key members (thus a range of perspectives and backgrounds) and the overarching plan to ‘create together’. How to achieve this, however, was to be developed together along the way, following a deliberately dynamic process.

Very ‘structured unstructured’. That there is a clearly communicated point in it being unclear right now. And that we chose this path so that we could find our way together.

(I4, Round 1)

The explorative process in co‐creation was expressed as ‘finding and understanding where we are’ (Round 1) and ‘searching for’ and ‘testing’ a structure (Round 2). Doing so required ‘calibrating expectations’ (Round 4) to ensure everyone agreed on both the journey and the destination. One participant also pointed out the importance of compromise, that although you might discuss and agree on the overall process, it was ‘the nature of [co‐creating] things’ to not always get exactly what you wanted (I1, Round 4).

Another aspect of dynamic co‐creation that participants noted was roles and engagement. They described how roles could shift as members collaborated in various constellations, such as within the management group or among the co‐authors of a specific study. For example, patient innovators could become researchers since they are equal members in the co‐creative research process.

That in one meeting I'm a participant … in another meeting maybe I'm leading something. In a third meeting I'm not even involved because I don't think it has anything to do with me. So, I'm completely okay if everyone isn't involved in everything. Or if they haven't signed off on all of our content.

(I1, Round 3)

There was some discrepancy between views on member engagement. This was more clearly articulated later in the partnership programme. Several participants expressed concern early on that striving to co‐create everything might entail everyone needing to be involved in everything (each step of each project, e.g.), a process that seemed impossible due to the group's size and diverse backgrounds. Participants soon realized that everyone could not be involved in everything. Being ‘pragmatic’ was identified as a key co‐creation characteristic.

[Co‐creation] is about participation and empowerment and that there are some things happening at the same time that not everyone can be involved with or do, and that's where you need to find some form of, like, pragmatism in it.

(I1, Round 1)

This concept of exploration and flexibility was present throughout all interview rounds. However, the last interviews included more examples of what had been co‐created on a project level, including everything ‘from defining the research question to actually doing part of the research together’ (R5, Round 4).

### 3.3 Striving for Equity, Not Equality

This theme focuses on the importance of ensuring equitable participation among all members. Co‐creation was described as ‘inclusive’, meaning that it did not exclude the patient innovators from the programme activities. Instead, co‐creation was described as letting people with lived experience control a large part of the work, including the research.

The [co‐creation of the] Patient in the ‘Driver's Seat programme’ shouldn't only apply to the innovations but should also apply to the research.

(R8, Round 2)

Co‐creation was understood as non‐hierarchical, where, although the patient innovators provide the starting point, everyone participates on ‘equal terms’. All perspectives are taken into account and things are done together regardless of role or background. One participant said that in co‐creation ‘all roles are equally important and that you do your bit, but it does not mean that one thing is more important than another’ (I7, Round 4). Another described it as

That you listen, and that there's a respect for all of the different competencies that we have, because if it's co‐creation then it must be on equal terms. That's what co‐creation is.

(R12, Round 4)

Co‐creation was also described as ‘a feeling of ownership, co‐ownership’ and ‘co‐responsibility, a commitment by the various parties’. According to the participants, co‐creation is what follows after the agreement of core goals, even if there continued to be individual agendas. It was emphasized that the patient innovators are not only there to represent their innovations.

They don't want to get run over, so to speak. They want to be involved in the creation of the research questions and the same with the research process and maybe even in the analysis of the data, to some extent at least.

(R11, Round 2)

As one participant pointed out, everyone was invited to contribute, but ‘someone has to take command’ (R11, Round 4). The same participant described the patient innovators as ‘gatekeepers’ to the innovations and their respective user communities, the patient groups and the healthcare system. It was raised that there were unavoidable inequities, such as the greater number of researchers compared to patient innovators in the group and the fact that the researchers often worked more hours in the programme than the patient innovators. However, as several participants confirmed, as long as researchers confirmed their work with the patient innovators, it was still considered co‐creation.

Another unavoidable inequity in the partnership programme was that the programme leaders (PIs) were researchers. Several participants felt that a conscious shift of power in favour of the patient innovators was thus needed to level the playing field. Such unevenness within the programme meant that the focus was more on equitable participation rather than equal conditions. As one researcher said,

As a researcher you have to put yourself in the passenger seat so that you can somehow achieve equal terms and conditions because I think the tradition, the culture … it is so engrained somehow, and I know that initially there was a ton of discussion around that.

(R16, Round 4)

### 3.4 Diverse Value‐Creation, Tangible and Intangible

Lastly, co‐creation is understood as generating something of value. Participants expressed tangible ‘outputs’ of value (outcomes) for the innovations included in the partnership programme and for research in the form of scientific publications. Participants also expressed the acquisition of intangible values in the form of learning on an individual and larger scale. The respondents felt that the work they were doing would not be co‐creation if it merely ended when the programme ended. Lasting co‐creation ‘should lead to something bigger’.

#### 3.4.1 Tangible: Research and Innovation

As already touched upon in earlier themes, co‐creation is understood as more patient‐centric and relevant than traditional research methods. The research undertaken by the partnership programme was based on practical needs expressed by patients and aimed to contribute to the work of the patient innovators. Participants described the value of co‐creation on a meta‐level. Co‐creation not only contributed to their knowledge of co‐creation but also showed them how to do co‐creation and how to do it in the right way. Through the development of knowledge of co‐creation, including the discussion of different roles, the overall aim was to

Renew healthcare with the clear starting point being the patients' or informal caregivers' needs and situations and ideas.

(R5′, Round 2)

Comments in the first two interview rounds focused on the co‐creation of relevant research, mainly to support the innovations included in the partnership programme and healthcare in general. In the third round, participants started to describe co‐creation in more general terms, as a process that could generate any number of outputs.

It's possible to co‐create a product, e.g., or a service. Co‐creative research is more like narrow, a narrower term. It's about co‐creation but within research. So co‐creation is broader.

(R11, Round 3)

According to participants, co‐creation is more than just the production of research publications. Co‐creative research is understood to produce more ‘relevant’ research ‘closer’ to patients' reality.

We co‐create how all [of the partnership programme] functions. And what it leads to. But it's bigger than just getting eight papers published. It's a co‐creation between colleagues and between experiences.

(R17, Round 4)

As the innovations were patient‐led and aimed at filling a void in the healthcare system, further developing them was seen as a way of creating a lasting impact. The co‐creative efforts involved in this process are not just research, they are something more.

You need to try to reach some sort of improvement of course. At the same time as doing research, though, there is something else happening … Co‐created research is then a bit like co‐creation; it isn't just research, it's something more than research.

(I3, Round 4)

#### 3.4.2 Intangible: Learning

As for intangible value creation, participants consistently described learning as a key part of co‐creation. According to one participant, the learning they had experienced would, in turn, lead to better work processes and outcomes. Since co‐creation was seen as explorative, a learning approach was also emphasized. The first round of comments focused on programme‐level learning and developing the co‐creation process within the group, whereas in later rounds participants described more of their own personal development through participating in research and ‘showing consideration to each other's experiences’.

It's partly that I'm learning new things, but it's also about getting new perspectives and … yeah, that I'm also able to develop because of the programme. There's also a hope I have. Besides the hope that our work will lead … to something bigger for the healthcare system, I'd like to think that I'll come out of this, somehow, enriched by this experience.

(R5, Round 2)

## 4 Discussion

**Figure 1:**
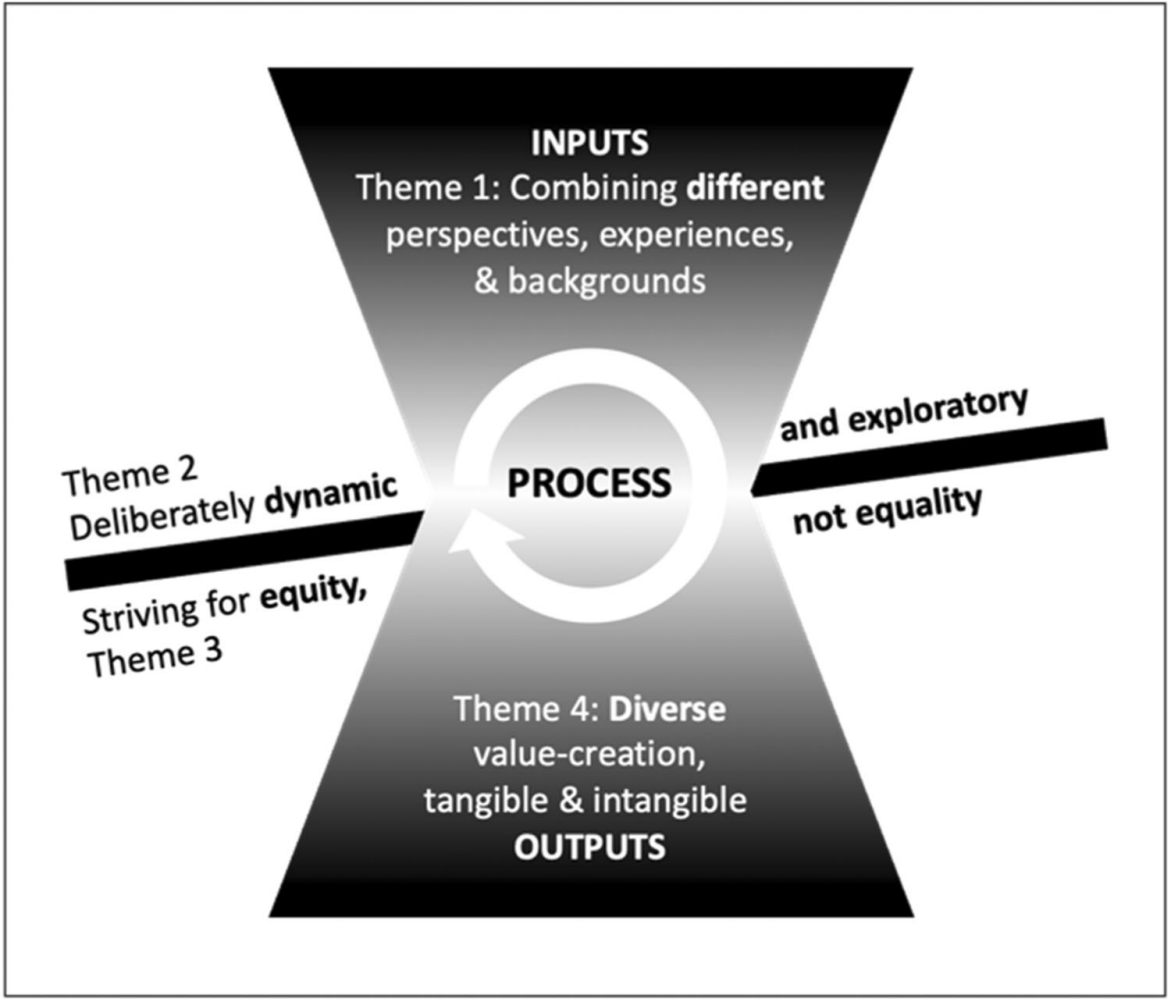
Schematic illustration of the complementary nature of the four understandings (themes) of co‐creation as diverse components of a co‐creation programme that evolves over time: ‘inputs’ are processed into ‘outputs’.

This study explored how members of a partnership research programme on patient‐driven innovations understand co‐creation in the research process and if this understanding changed during their participation in the programme. Although we did not detect any evident conceptual changes over time, we did identify four distinct understandings of co‐creation, which we then organized into four themes reflecting various understandings of co‐creation. The fact that these themes appeared to demonstrate shifts in their relationships with each other suggests that there may be different phases or cycles of understanding in a co‐creation programme. This suggests that the variations in understanding that we observed do not necessarily reflect different understandings of co‐creation in research but rather four complementary understandings of it (Figure 1).

The findings of this study suggest that the four co‐creation themes can be instrumentally conceptualized using the different phases of a research programme: the *inputs* (resources), the *process* (activities) and the *outputs*. Many existing co‐creation frameworks have used similar representations [9, 18, 27]. In this study, *inputs* represent the different perspectives that the partners bring to the table. *Process* represents how partners collaborate by bringing together the *inputs*, combining them in a deliberately dynamic and explorative approach, while also striving for equity between differing backgrounds and competences. Lastly, *outputs* represent how the creation of tangible and intangible value resulting from the *inputs* and *process*.

### 4.1 Inputs—Different Perspectives

Situated in the specific context of a partnership programme with researchers and patient innovators, the findings show that members contributed different perspectives, experiences and backgrounds as their input to the co‐creation process. This view is also central to many action‐ and participatory‐based research approaches [14]. According to the present study, perspectives must be mixed and combined to fulfil the aim of the partnership programme. This view is the foundation for interdisciplinary and translational research but also for innovation processes that aim to address and tackle grand societal challenges, where knowledge and experiences from different disciplines and sectors must meet and collaborate [28]. Integrating various perspectives and stakeholders is considered central to health innovation. Nevertheless, a study of health innovation networks could find no evidence of active end‐user or patient participation [29]. This suggests that the identified value of combining different perspectives is, in fact, not easily translated into practice.

### 4.2 Process—Dynamic and Explorative, Striving for Equity

Understandings of co‐creation also engage with the *process* of how research is carried out. In the studied context of a research partnership with patient innovators, this understanding describes the *process* of co‐creation in research as a balance between flexible and structured. Co‐creation was understood as an intentionally open, explorative and malleable process. This aligns with existing research on formalized structures or processes as facilitators for co‐creation and how formal rules can decrease uncertainty and enhance perceived abilities. The findings also align with the importance of an adaptive and developmental process in co‐creation. Co‐creation has a dynamic nature with nonlinear causation chains [8], and the co‐creation process requires an exploratory space and a generative process that can lead to different *outputs* [30].

The understanding of co‐creation also involves an awareness of the *process* of equitable treatment. The findings of this study illustrate how participants were conscious of the need for balance between treating all members the same and letting all members take the lead. This balancing of sometimes almost contradictory understandings might relate to the findings of Pearce et al. [31], who, in their study of co‐creation between researchers, industry and users, identified trust as an essential element of success.

Alongside inclusivity, this study also identified co‐ownership and co‐responsibility as other aspects of co‐creation. The existing literature identifies different types and modes of co‐creation, depending on the level of engagement, from an informant and recipient to an endorser, commissioner and, finally, a co‐researcher [10]. The understanding of co‐creation described by the patient innovators and researchers in this study does not view participation in such hierarchical terms at all. As they understand it, co‐creation involves equity, co‐ownership and co‐responsibility, all concepts that point to the idea that co‐creation engages co‐researchers and nothing less.

### 4.3 Output—Diverse Value Creation

Lastly, the understanding of co‐creation includes diverse value creation as an *output*. This implies that research is viewed as more patient‐centric and practically relevant than other research approaches, specifically because it involves patient innovators as stakeholders. Thus, in this study, the *outputs* were seen as having both scientific and practical value. Value creation is central to many co‐creation approaches, specifically the value of actionable and useable research findings. Scholars argue that co‐creation between researchers and practitioners bridges a gap between research and practice [31] and enables societal impact [8]. Studies show how practitioners [10] and industry [15] can engage with researchers to address practical problems that can lead to both practical solutions and scientific publications. In our study, actionable research was understood as research that yields more than scientific publications; it also contributed to innovation development. In their study of a strategic approach to innovation, Frow et al. [16] argue that co‐creation offers companies and collaborators significant opportunities for innovation because each partner provides access to new resources. In a study on third‐sector organizations and the co‐production of health innovations, Windrum [29] point out that, despite the rhetoric of European health policies about patient participation, patients are still absent from the co‐production of health innovations. The approach chosen in the programme studied here shows that this trend can be broken by putting patients in the driver's seat.

### 4.4 Implications for Practice

The aim of this study was to explore co‐creation within the specific context of a research partnership programme. The findings, illustrating patient innovators' and researchers' understanding of co‐creation, could intuitively be mapped onto a timeline representing the programme's progression. While the representation of co‐creation as inputs, process and outputs is already common in the literature, the findings of this study can complement existing models by facilitating discussions among members of co‐creating groups in planning, carrying out or evaluating co‐creation efforts. These discussions can be guided by asking questions addressing the different phases, such as:

*Inputs*: Which different perspectives, experiences and backgrounds are needed/involved/missing in our project?
*Process*: Is our process sufficiently dynamic and exploratory? How can/do we ensure equity among members?
*Outputs*: What types of value creation do we anticipate? What outcomes have we achieved?

### 4.5 Strengths and Limitations

One of this study's main strengths was its longitudinal design. Data were collected over a 3‐year period, which allowed us to analyse participants' views over time. However, as the members of the studied partnership programme varied across time, as members joined or left the programme, the interview participation also varied. Not all participants were interviewed multiple times.

The authors' involvement as study participants may be viewed as both a strength and a limitation. On the one hand, being ‘native researchers’ [5] could benefit the interpretation of findings with authentic inside perspectives. On the other hand, insufficient detachment from the data could risk introducing bias. In particular, as only participants with roles as researchers co‐authored this publication, there was a risk of bias favouring the researchers' perspectives. The member‐checking workshop, which involved patient innovators, contributed to strengthening reflexivity by combining different perspectives in the discussion of findings. Nevertheless, we acknowledge that the involvement of a patient innovator as a co‐author of this publication would have been desirable. Regarding the transferability of our findings, we believe that other researchers and patient innovators may find recognition in the meanings of co‐creation that our study describes. Similarly, the proposed framework is generic enough to suit various co‐creation projects. Nevertheless, our ambition was not to propose one model that fits all. Different contexts will likely bring complementary, and possibly diverging, perspectives and nuances to co‐creation. However, since the authors were involved in interpreting findings, the benefit of authenticity against the lack of separation from the data needs to be thoroughly weighed. The reflexive approach to thematic analysis supported the authors in representing all perspectives when discussing and reporting the findings.

## 5 Conclusion

Our study identified four distinct yet complementary understandings of co‐creation from the perspectives of patient innovators and researchers. These understandings emphasize the importance of combining different perspectives, experiences and backgrounds when initiating a co‐creation programme; that co‐creation should occur in a deliberately dynamic and exploratory manner and should strive for equity, not equality, among members; and that co‐creative efforts should anticipate both tangible and intangible value creation. Our findings can be used to guide planning discussions within co‐creative groups and support formative and summative evaluation processes.

## Author Contributions

**Hanna Jansson:** conceptualization, methodology, writing–original draft, writing–review and editing, data curation, visualization, validation. **Jamie L. Luckhaus:** methodology, writing–original draft, writing–review and editing, data curation, investigation, validation. **Henna Hasson:** conceptualization, methodology, writing–review and editing, funding acquisition. **Pamela Mazzocato:** methodology, writing–review and editing. **Terese Stenfors:** conceptualization, methodology, writing–review and editing. **Carolina Wannheden:** methodology, writing–review and editing.

## Ethics Statement

Ethical approval for the study was obtained from the Swedish Ethical Review Authority (Reg. No. 2019‐03849).

## Conflicts of Interest

All authors, except Jamie L. Luckhaus, are members of the research programme that was studied.

## Supporting information

Supporting information.

Supporting information.

Supporting information.

## Data Availability

The data that support the findings of this study are available on request from the corresponding author. The data are not publicly available due to privacy or ethical restrictions.
