# Supplementary material for: Understanding Co‐Creation in a Research Partnership Programme Exploring Patient‐Driven Innovations: A Qualitative Longitudinal Study
Source: Health Expect. 2024 Aug 30;27(5):e70003. doi: 10.1111/hex.70003 (PMC11362650; doi:10.1111/hex.70003)
Supplement: Supplementary file 1 — Supporting information. [file HEX-27-e70003-s003.docx]

# Appendix A: Interview guide

Extract of the interview guides relevant for the study.

| **Interview question** | **Interview round^1^** | | | |
| --- | --- | --- | --- | --- |
|  | **1** | **2** | **3** | **4** |
| You may start by telling a bit about how you joined the programme (for new participants; if not new, start with question 2). | X | x | x | x |
| What is your role in [name of the partnership programme]? | x | x | x | x |
| How would you describe the current phase of the programme? | x | x | x | x |
| Do you see any potential challenges with the programme? | x | x | x | x |
| What are your expectations for the programme? Have your expectations changed? | x | x | x | x |
| [Name of the partnership programme] is a programme that aims to be co-creative. What does that mean to you? | n/a | n/a | X | x |
| How do you perceive the current conditions for co-creation within the programme? | x | x | x | x |
| What do you think can be expected from a co-creative research programme, the short-term and long-term effects? | x | x | x | x |
| Is there anything in general that you think could have been done differently in the programme? If so, what and in what way? | x | x | x | x |
| What have you learned during this process? | n/a | n/a | x | X |
| Is there anything you think I should have asked but didn’t? Is there anything you would like to add before we conclude? | x | x | x | x |

^1^ x (lowercase) indicates that the question area was addressed, X (uppercase) indicates that more emphasis was put on the question area (e.g., by asking follow-up and probing questions), and n/a indicates that it was not addressed.
