# Supplementary material for: Understanding Co‐Creation in a Research Partnership Programme Exploring Patient‐Driven Innovations: A Qualitative Longitudinal Study
Source: Health Expect. 2024 Aug 30;27(5):e70003. doi: 10.1111/hex.70003 (PMC11362650; doi:10.1111/hex.70003)

Appendix B: Member checking workshop

An example from the member checking workshop. The figures below represent the initial themes from interview round 3 prior to the member checking exercise (A), the themes that were developed by two individual groups based on interview extracts that they were provided from interview round 3 (B and C), and the final combined themes after comparing and merging the themes from all interview rounds (D).

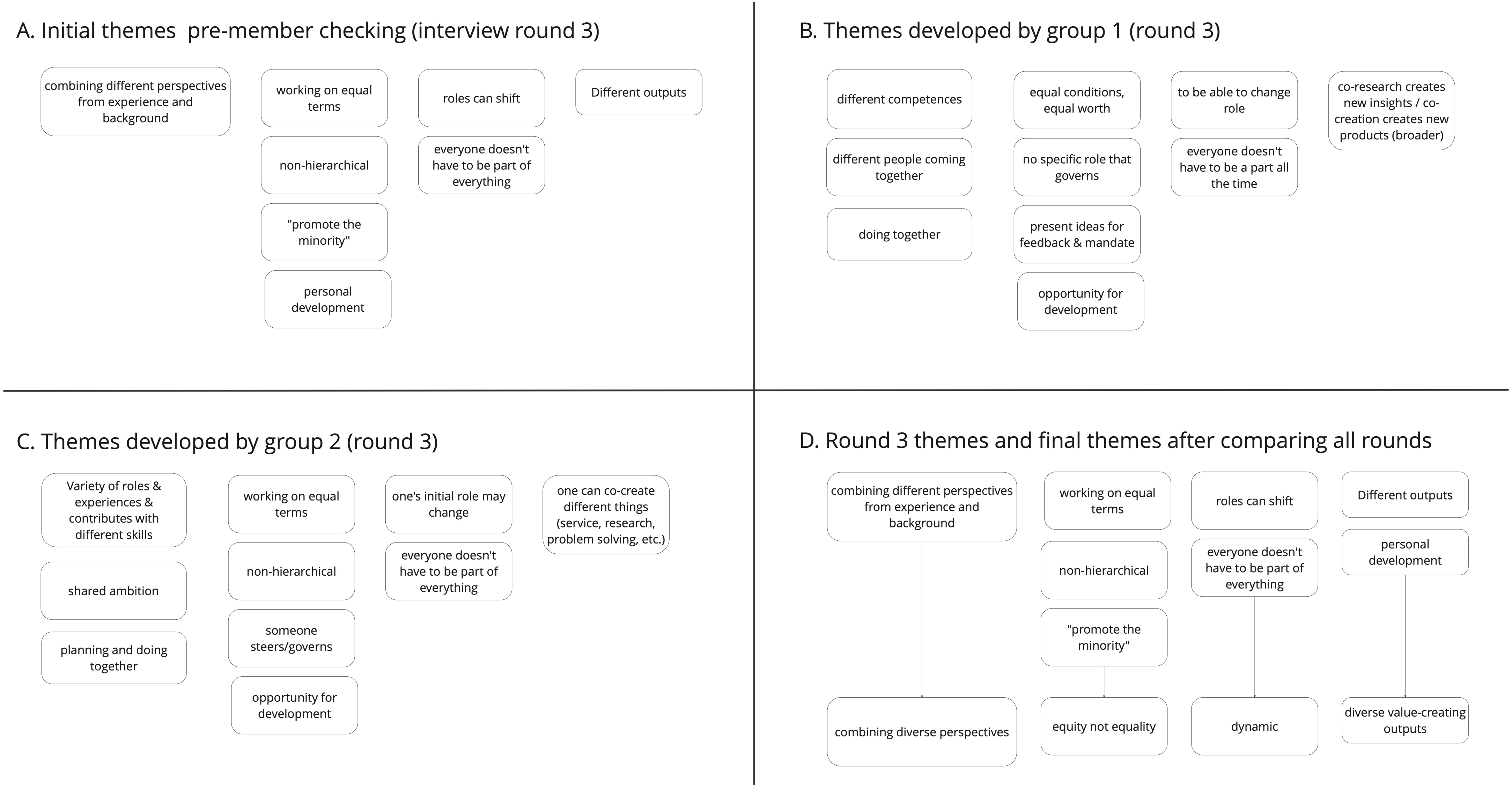

Supplement: Supplementary file 2 — Supporting information. [file HEX-27-e70003-s001.pdf]
