# Supplementary figures and images for: Understanding Co‐Creation in a Research Partnership Programme Exploring Patient‐Driven Innovations: A Qualitative Longitudinal Study
Source: Health Expect. 2024 Aug 30;27(5):e70003. doi: 10.1111/hex.70003 (PMC11362650; doi:10.1111/hex.70003)

# Appendix C: Comparing and combining interview rounds

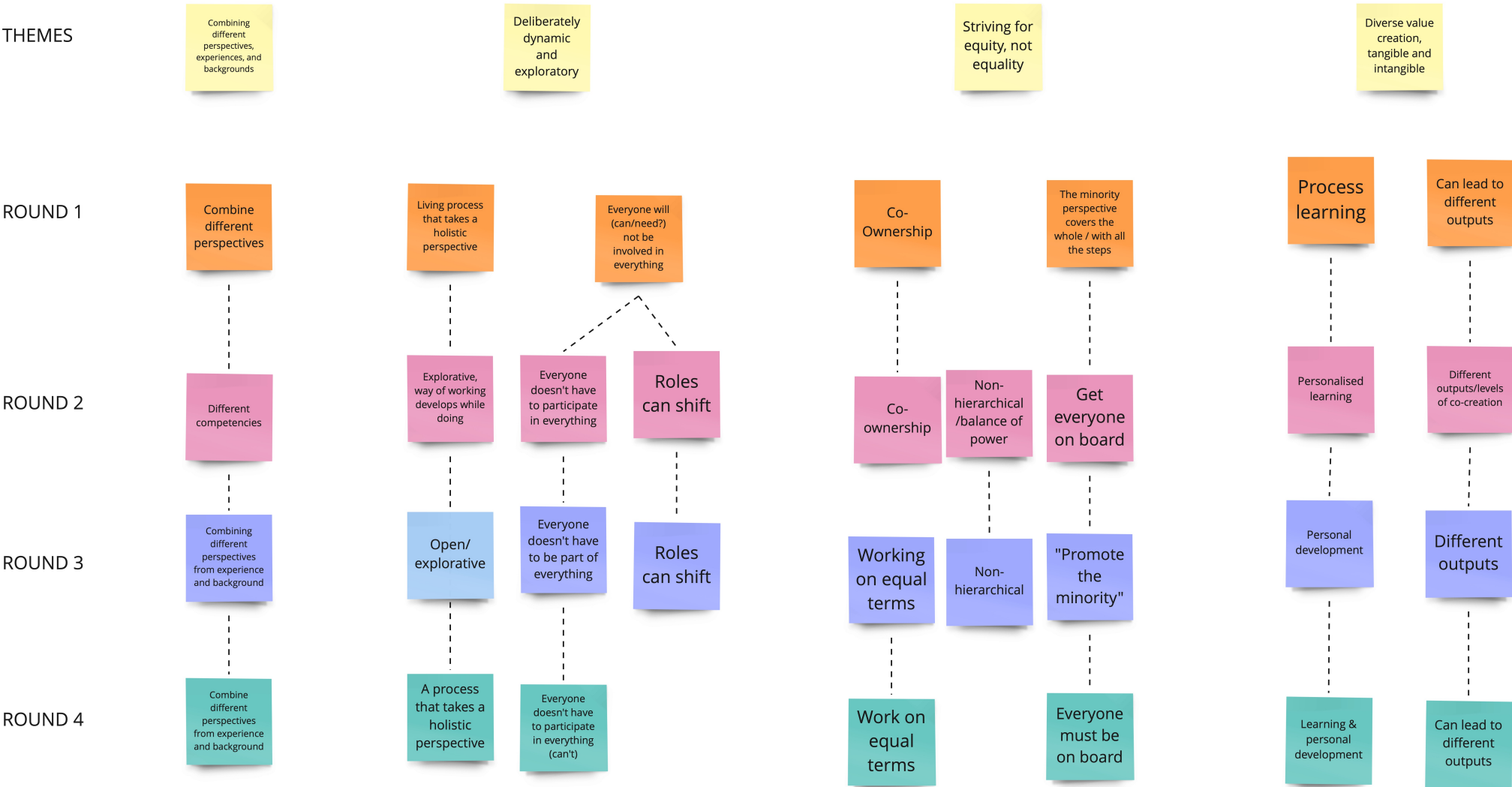

Supplement: Supplementary file 3 — Supporting information. [file HEX-27-e70003-s002.pdf]
